# Supplementary material for: The impact of family environment on self-esteem and symptoms in early psychosis
Source: PLoS One. 2021 Apr 5;16(4):e0249721. doi: 10.1371/journal.pone.0249721 (PMC8021173; doi:10.1371/journal.pone.0249721)
Supplement: S1 Fig — (DOCX) [file pone.0249721.s001.docx]

**RELATIVES OF EARLY PSYCHOSIS PATIENTS**

**Figure S1.** Flow chart describing the participants included in the study

**INCIPIENT PSYCHOSIS PATIENTS**

*Relatives were referred to the study*

*by their respective affected family*

*members (i.e., patients who were*

*already participating in the study).*

Invited to participate in the study

N=179 (105 of ARMS and 74 of FEP)

Invited to participate in the study

N=208 (111 ARMS and 97 FEP)

Refused to participate:

N= 39 (24 of ARMS and 15 of FEP)

Participants of the pilot study:

N=18 (6 of ARMS and 12 of FEP)^1^

Refused to participate:

N= 35 (17 ARMS and 18 FEP)

Excluded for not meeting inclusion criteria: N=24 (7 ARMS and 17 FEP)

Participants of the pilot study:

N=21 (7 of ARMS and 14 of FEP)^1^

Excluded from the analyses due to missing data on some core measures of the study^3^

N= 35 (20 ARMS and 15 FEP)

**Relatives of early psychosis patients at BASELINE**

N=122 (75 of ARMS and 47 of FEP)^2^

**Early psychosis patients at BASELINE**

N=128 (80 ARMS and 48 FEP)

**Total of patient-relative**

**DYADS at BASELINE**

N=92 (58 of ARMS and 34 of FEP)

Excluded from the analyses due to missing data on some core measures of the study^5^

N= 34 (21 of ARMS and 13 of FEP)

Excluded from the analyses due to missing data on some core measures of the study^4^

N= 15 (8 of ARMS and 7 of FEP)

**Final included**

**EARLY PSYCHOSIS DYADS**

N=58 (37 of ARMS and 21 of FEP)

**Final included**

**EARLY PSYCHOSIS DYADS**

N=77 (50 of ARMS and 27 of FEP)

**Final included**

**EARLY PSYCHOSIS PARTICIPANTS**

N=93 (60 ARMS and 33 FEP)

**GOALS 2 and 4**

**GOAL 1**

**GOAL 3**

^1^ Patients as well as relatives participating in a pilot study of this project were not included in the present study because they completed a less comprehensive assessment at baseline.

^2^ Two types of relatives were included at baseline: the key relatives, those who were included in the present study (N=92) and the second closest relative (N=30).

^3^ Brief Dyadic Scale of Expressed Emotion (BDSEE), Rosenberg Self-Esteem Scale (RSES) or Positive and Negative Syndrome Scale (PANSS).

^4^ Family Questionnaire (FQ), RSES or PANSS.

^5^ FQ, BDSEE, RSES, PANSS.
