# Supplementary material for: The impact of family environment on self-esteem and symptoms in early psychosis
Source: PLoS One. 2021 Apr 5;16(4):e0249721. doi: 10.1371/journal.pone.0249721 (PMC8021173; doi:10.1371/journal.pone.0249721)
Supplement: S1 Table — (DOCX) [file pone.0249721.s002.docx]

**Table S1. Pearson correlations among BDI, CDS and PANSS-Depression (Sample 2; n=58).**

|  | **BDI** | **CDS** | **PANSS-Depression** |
| --- | --- | --- | --- |
| **BDI** | **-** | 0.78*** | 0.73*** |
| **CDS** | 0.78*** | - | 0.81*** |
| **PANSS-Depression** | 0.73*** | 0.81*** | - |

BDI: Beck Depression Inventory, CDS: Calgary Depression Scale, PANSS: Positive and Negative Syndrome Scale.

*** p<0.001.

***Principal component analysis (PCA) of the composite measure of depression***

A PCA was conducted on the BDI-II, the CDS, and the “depression” item from the PANSS (item G6) to calculate a general measure of depression. Results of Kaiser-Meyer-Olkin measure (KMO = 0.89) of sampling adequacy and Bartlett's test of sphericity (χ2 [465] = 1795, *p* < 0.001) revealed that our application of factor analysis was valid in the study sample. Examination of eigenvalues indicated 7-factor solution explaining 64.5% of the variance. However, scree plot suggested the presence of a principal depression factor, and component matrix indicated that 27 of the 31 total items had their highest factor load on the first factor (all > 0.48). Items that loaded < 0.40 in the principal depression factor were: BDI-II “Agitation” (0.38) and “Loss of interest in sex” (0.36), CDS “Guilty ideas of reference” (0.39) and “Early wakening” (0.26). Thus, factor score coefficients of the first factor were calculated using the Regression method and employed as a variable representing a composite measure of depression.
