# Supplementary material for: The impact of family environment on self-esteem and symptoms in early psychosis
Source: PLoS One. 2021 Apr 5;16(4):e0249721. doi: 10.1371/journal.pone.0249721 (PMC8021173; doi:10.1371/journal.pone.0249721)
Supplement: S2 Table — (DOCX) [file pone.0249721.s003.docx]

**Table S2. Pearson correlations among BDI, CDS and PANSS-5 Factors-Depression/Anxiety Scale (Sample 2; n=58).**

|  | **BDI** | **CDS** | **PANSS-5 Factors**  **Depression/Anxiety Scale** |
| --- | --- | --- | --- |
| **BDI** | **-** | 0.78*** | 0.65*** |
| **CDS** | 0.78*** | - | 0.75*** |
| **PANSS-5 Factors**  **Depression/Anxiety Scale** | 0.65*** | 0.75*** | - |

BDI: Beck Depression Inventory, CDS: Calgary Depression Scale, PANSS: Positive and Negative Syndrome Scale.

*** p<0.001.

***Principal component analysis (PCA) of the composite measure of negative affect (NA)***

A PCA was conducted on the BDI-II, the CDS, and the “Depression/Anxiety” factor from the PANSS- Five Factors (Emsley, 2003) to calculate a general measure of negative affect. Results of Kaiser-Meyer-Olkin measure (KMO = 0.88) of sampling adequacy and Bartlett's test of sphericity (χ2 [595] = 2030, *p* < 0.001) revealed that our application of factor analysis was valid in the study sample. Examination of eigenvalues indicated 8-factor solution explaining 64.9% of the variance. However, scree plot suggested the presence of a principal negative affect factor, and component matrix indicated that 30 of the 35 total items had their highest factor load on the first factor (all > 0.42). Items that loaded < 0.40 in the principal depression factor were: BDI-II “Agitation” (0.39) and “Loss of interest in sex” (0.37), CDS “Guilty ideas of reference” (0.39), and “Early wakening” (0.26), and PANSS “Somatic concern” (0.07). Thus, factor score coefficients of the first factor were calculated using the Regression method and employed as a variable representing a composite measure of NA, encompassing a wide variety of affective-related symptoms.
