# Supplementary material for: The impact of family environment on self-esteem and symptoms in early psychosis
Source: PLoS One. 2021 Apr 5;16(4):e0249721. doi: 10.1371/journal.pone.0249721 (PMC8021173; doi:10.1371/journal.pone.0249721)
Supplement: S3 Table — (DOCX) [file pone.0249721.s004.docx]

**Table S3. Descriptive data on socio-demographic characteristics of early psychosis patients and their respective relatives.**

|  | **Sample 1**^a^ | | **Sample 2**^b^ | | **Sample 3**^c^ |
| --- | --- | --- | --- | --- | --- |
|  | ***Patients*** | ***Relatives*** | ***Patients*** | ***Relatives*** | ***Patients*** |
|  | **n (%)** | **n (%)** | **n (%)** | **n (%)** | **n (%)** |
| **Age** *(mean, SD)* | 21.96 (4.6) | 50.71 (10.8) | 22.05 (4.6) | 50.69 (11.3) | 22.28 (4.4) |
| **Gender** |  |  |  |  |  |
| Males | 54 (70.1) | 17 (22.1) | 42 (72.4) | 10 (17.2) | 64 (68.8) |
| Females | 23 (29.9) | 60 (77.9) | 16 (27.6) | 48 (82.8) | 29 (31.2) |
| **Ethnicity** |  |  |  |  |  |
| Western Europeans | 60 (77.9) | 62 (80.5) | 46 (79.3) | 47 (81.0) | 70 (75.3) |
| Eastern Europeans | 1 (1.3) | 3 (3.8) | 1 (1.7) | 3 (5.1) | 2 (2.2) |
| Asians | 1 (1.3) | 3 (3.8) | 1 (1.7) | 3 (3.4) | 2 (2.2) |
| Latin Americans | 11 (14.3) | 9 (11.9) | 7 (12.1) | 5 (10.5) | 14 (15.1) |
| Mixed | 4 (5.2) | - | 3 (5.2) | - | 5 (5.4) |
| **Occupation** |  |  |  |  |  |
| Unemployed/unoccupied | 29 (37.7) | 25 (32.5) | 22 (37.9) | 20 (34.5) | 37 (39.8) |
| Employed | 10 (13.0) | 46 (59.7) | 7 (13.8) | 36 (62.1) | 11 (11.8) |
| Student | 32 (41.5) | 1 (1.3) | 25 (43.1) | 1 (1.7) | 41 (44.1) |
| Sick leave/community rehabilitation | 6 (7.8) | 5 (6.5) | 3 (5.2) | 1 (1.7) | 4 (4.3) |
| **Marital Status** |  |  |  |  |  |
| Single | 73 (94.8) | 2 (2.6) | 56 (96.6) | 2 (3.4) | 89 (95.6) |
| Married or analogous | 3 (3.9) | 48 (62.3) | 1 (1.7) | 35 (60.3) | 2 (2.2) |
| Separated/divorced/widowed | 1 (1.3) | 27 (35.1) | 1 (1.7) | 21 (36.3) | 2 (2.2) |
| **Relationship to patient** |  |  |  |  |  |
| Father | - | 11 (14.3) | **-** | 5 (8.7) | **-** |
| Mother | - | 58 (75.3) | **-** | 47 (81.0) | **-** |
| Other | - | 8 (10.4) | **-** | 6 (10.3) | **-** |
| **Living with patient** |  |  |  |  |  |
| Yes | - | 69 (89.6) | **-** | 52 (89.7) | **-** |
| No | - | 8 (10.4) | **-** | 6 (10.3) | **-** |
| **Frequency of contact** |  |  |  |  |  |
| Between 1 and 14h a week | - | 27 (35.2) | **-** | 20 (34.5) | **-** |
| Between 15 and 27h a week | - | 18 (23.4) | **-** | 14 (24.1) | **-** |
| $\leq$ 28h a week | - | 31 (40.4) | **-** | 22 (41.4) | **-** |

^a^ n =77 early psychosis patients and their respective relatives

^b^ n =58 early psychosis patients and their respective relatives

^c^ n= 93 early psychosis patients
