# Supplementary material for: The impact of family environment on self-esteem and symptoms in early psychosis
Source: PLoS One. 2021 Apr 5;16(4):e0249721. doi: 10.1371/journal.pone.0249721 (PMC8021173; doi:10.1371/journal.pone.0249721)
Supplement: S4 Table — (DOCX) [file pone.0249721.s005.docx]

**Table S4. Descriptive data of early psychosis patients and their respective relatives.**

|  |  | **Sample 1**^a^ | | | **Sample 2**^b^ | | | **Sample 3**^c^ | | |
| --- | --- | --- | --- | --- | --- | --- | --- | --- | --- | --- |
|  | Possible Score Range | α | Observed Score  Range | *Mean (SD)* | α | Observed Score  Range | *Mean (SD)* | α | Observed Score  Range | *Mean (SD)* |
| **Relatives’ EE (FQ)** |  |  |  |  |  |  |  |  |  |  |
| Criticism | 10-40 | 0.88 | 10-36 | 20.97(6.53) | 0.87 | 11-36 | 20.81(6.17) | - | - | - |
| EOI | 10-40 | 0.83 | 11-36 | 23.58(5.88) | 0.84 | 11-36 | 24.16(5.91) | - | - | - |
| **Perceived EE (BDSEE)** |  |  |  |  |  |  |  |  |  |  |
| Perceived Criticism | 10-40 | - | - | - | - | - | - | 0.85 | 4-37 | 17.92(9.09) |
| Perceived EOI | 10-60 | - | - | - | - | - | - | 0.80 | 6-51 | 25.72(12.09) |
| Perceived Warmth | 10-40 | - | - | - | - | - | - | 0.90 | 7-40 | 30.80(8.35) |
| **Self-esteem (RSES)** |  |  |  |  |  |  |  |  |  |  |
| Positive SE | 0-15 | 0.86 | 1-15 | 8.47(3.27) | 0.86 | 1-15 | 8.50(3.32) | 0.84 | 1-15 | 8.42(3.27) |
| Negative SE | 0-15 | 0.81 | 0-15 | 7.88(3.62) | 0.81 | 0-15 | 7.90(3.72) | 0.81 | 0-15 | 8.08(3.65) |
| Global SE | 0-30 | 0.88 | 1-30 | 15.58(6.30) | 0.89 | 1-30 | 15.60(6.46) | 0.88 | 1-30 | 15.34(6.32) |
| **Positive Symptoms (PANSS)** | 7-49 | - | 7-24 | 12.87 (3.44) | - | 7-19 | 12.19(2.90) | - | 7-22 | 12.81(3.32) |
| **Paranoia (PANSS)** | 1-7 | - | 1-5 | 2.77(1.16) | - | 1-5 | 2.72(1.09) | - | 1-5 | 2.87(1.07) |
| **Patients’ Distress** |  |  |  |  |  |  |  |  |  |  |
| Anxiety (PANSS) | 1-7 | - | - | *-* | - | 1-5 | 2.97(1.09) | *-* | *-* | *-* |

SD: Standard Deviation; FQ: Family Questionnaire; BDSEE: Brief Dyadic Scale of Expressed Emotion; RSES: Rosenberg Self-Esteem Scale; PANSS: Positive and Negative Syndrome Scale; EOI: Emotional Over-Involvement; SE: Self-Esteem.

^a^ n =77 early psychosis patients and their respective relatives

^b^ n =58 early psychosis patients and their respective relatives

^c^ n= 93 early psychosis patients.
