# Supplementary material for: The impact of family environment on self-esteem and symptoms in early psychosis
Source: PLoS One. 2021 Apr 5;16(4):e0249721. doi: 10.1371/journal.pone.0249721 (PMC8021173; doi:10.1371/journal.pone.0249721)
Supplement: S5 Table — (DOCX) [file pone.0249721.s006.docx]

**Table S5. Pearson correlations of relatives’ EE with patients’ symptoms (Sample 1; n=77).**

|  | **Relatives’ EE** | |
| --- | --- | --- |
|  | **Relatives’ Criticism** | **Relatives’ EOI** |
| **Patients’ symptoms (PANSS)** |  |  |
| Positive symptoms | 0.12 | 0.04 |
| Paranoia | 0.04 | -0.05 |

EE: Expressed Emotion; EOI: Emotional Over-Involvement; PANSS: Positive and Negative Syndrome Scale.
