# Supplementary material for: The impact of family environment on self-esteem and symptoms in early psychosis
Source: PLoS One. 2021 Apr 5;16(4):e0249721. doi: 10.1371/journal.pone.0249721 (PMC8021173; doi:10.1371/journal.pone.0249721)
Supplement: S6 Table — (DOCX) [file pone.0249721.s007.docx]

**Table S6. Pearson correlations of patients’ SE with relatives’ EE and patients’ symptoms (Sample 1; n=77).**

|  | **Patients’ SE** | |
| --- | --- | --- |
|  | **Positive SE** | **Negative SE** |
| **Relatives’ EE (FQ)** |  |  |
| Criticism | -0.26* | 0.13 |
| EOI | -0.05 | -0.01 |
| **Patients’ symptoms (PANSS)** |  |  |
| Positive symptoms | -0.05 | 0.16 |
| Paranoia | -0.15 | 0.25* |

SE: Self-Esteem; EE: Expressed Emotion; FQ: Family Questionnaire; EOI: Emotional Over-Involvement; PANSS: Positive and Negative Syndrome Scale.

*p*<*0.05
