# Supplementary material for: The impact of family environment on self-esteem and symptoms in early psychosis
Source: PLoS One. 2021 Apr 5;16(4):e0249721. doi: 10.1371/journal.pone.0249721 (PMC8021173; doi:10.1371/journal.pone.0249721)
Supplement: S7 Table — (DOCX) [file pone.0249721.s008.docx]

**Table S7. Conditional indirect effects of relatives’ EE on symptoms through positive and negative SE (Sample 1; n=77).**

|  | | | **Conditional indirect effects at different values of the moderator** | | | | | | **Global Index of moderated mediation** | | | | |
| --- | --- | --- | --- | --- | --- | --- | --- | --- | --- | --- | --- | --- | --- |
|  |  |  |  |  |  |  | **95 % Bias-corrected**  **CI** | | |  |  | **95 % Bias-corrected**  **CI** | |
| **Predictor** | **Outcome** | **Mediator** | **Moderator** | **Level** | **Raw Parameter Estimate** | **SE** | **Lower** | **Upper** | **Index** | | **SE** | **Lower** | **Upper** |
| Relatives’ criticism | Positive symptoms | Positive SE | Group | ARMS | 0.010 | 0.022 | -0.011 | 0.095 | -0.123 | | 0.102 | -0.395 | 0.014 |
|  |  |  |  | FEP | -0.113 | 0.099 | -0.392 | 0.017 |  |  |  |  |  |
|  |  | Negative SE | Group | ARMS | 0.000 | 0.013 | -0.025 | 0.034 | 0.037 | | 0.069 | -0.038 | 0.309 |
|  |  |  |  | FEP | 0.038 | 0.068 | -0.031 | 0.332 |  |  |  |  |  |
| Relatives’ criticism | Paranoia | Positive SE | Group | ARMS | 0.006 | 0.009 | -0.004 | 0.040 | -0.036 | | 0.029 | -0.119 | 0.004 |
|  |  |  |  | FEP | -0.030 | 0.028 | -0.113 | 0.005 |  |  |  |  |  |
|  |  | Negative SE | Group | ARMS | 0.000 | 0.007 | -0.009 | 0.019 | 0.013 | | 0.023 | -0.015 | 0.086 |
|  |  |  |  | FEP | 0.013 | 0.022 | -0.011 | 0.097 |  |  |  |  |  |
| Relatives’  EOI | Positive symptoms | Positive SE | Group | ARMS | 0.002 | 0.018 | -0.021 | 0.066 | -0.012 | | 0.062 | -0.174 | 0.085 |
|  |  |  |  | FEP | -0.010 | 0.059 | -0.181 | 0.075 |  |  |  |  |  |
|  |  | Negative SE | Group | ARMS | -0.000 | 0.014 | -0.029 | 0.029 | -0.029 | | 0.069 | -0.238 | 0.057 |
|  |  |  |  | FEP | -0.029 | 0.068 | -0.234 | 0.051 |  |  |  |  |  |
| Relatives’ EOI | Paranoia | Positive SE | Group | ARMS | 0.001 | 0.007 | -0.009 | 0.026 | -0.004 | | 0.020 | -0.056 | 0.029 |
|  |  |  |  | FEP | -0.003 | 0.019 | -0.057 | 0.024 |  |  |  |  |  |
|  |  | Negative SE | Group | ARMS | -0.000 | 0.006 | -0.014 | 0.014 | -0.009 | | 0.023 | -0.079 | 0.023 |
|  |  |  |  | FEP | -0.009 | 0.022 | -0.078 | 0.019 |  |  |  |  |  |

Note: Results are based on 10,000 bias-corrected bootstrap samples.

^a^ Relatives’ Criticism, Relatives’ EOI (X-Independent variable) and Diagnostic Category (W-moderator) were mean centered prior to analysis.

*95% Confidence Interval does not include zero.
