# Supplementary material for: The impact of family environment on self-esteem and symptoms in early psychosis
Source: PLoS One. 2021 Apr 5;16(4):e0249721. doi: 10.1371/journal.pone.0249721 (PMC8021173; doi:10.1371/journal.pone.0249721)
Supplement: S8 Table — (DOCX) [file pone.0249721.s009.docx]

**Table S8. Pearson correlations of relatives’ EE with patients’ SE (Sample 2; n=58).**

|  | **Relatives’ EE** | |
| --- | --- | --- |
|  | **Relatives’ Criticism** | **Relatives’ EOI** |
| **Patients’ SE (RSES)** |  |  |
| Positive SE | -0.24^+^ | -0.02 |
| Negative SE | 0.16 | -0.004 |

EE: Expressed Emotion; EOI: Emotional Over-Involvement; RSES: Positive and Negative Syndrome Scale; SE: Self-Esteem.

^+^*p* <.10
