# Supplementary material for: The impact of family environment on self-esteem and symptoms in early psychosis
Source: PLoS One. 2021 Apr 5;16(4):e0249721. doi: 10.1371/journal.pone.0249721 (PMC8021173; doi:10.1371/journal.pone.0249721)
Supplement: S9 Table — (DOCX) [file pone.0249721.s010.docx]

**Table S9. Pearson correlations of patients’ perceived EE with relatives’ EE and patients’ SE (Sample 2; n=58).**

|  | **Patients’ perceived EE** | |
| --- | --- | --- |
|  | **Perceived criticism** | **Perceived EOI** |
| **Relatives’ EE (FQ)** |  |  |
| EE-Criticism | **0.40**** | 0.28* |
| EE- EOI | 0.27* | 0.28* |
| **Patients’ SE (RSES)** |  |  |
| Positive SE | -0.19 | -0.29* |
| Negative SE | **0.34**** | **0.46***** |

EE: Expressed Emotion; EOI: Emotional Over-Involvement; FQ: Family Questionnaire; RSES: Positive and Negative Syndrome Scale; SE: Self-Esteem.

*p*<*0.05; **p*≤* 0.01; *** p*<*0.001. Medium effect sizes (r ≥ 0.30) in bold.
