# Supplementary material for: The impact of family environment on self-esteem and symptoms in early psychosis
Source: PLoS One. 2021 Apr 5;16(4):e0249721. doi: 10.1371/journal.pone.0249721 (PMC8021173; doi:10.1371/journal.pone.0249721)
Supplement: S10 Table — (DOCX) [file pone.0249721.s011.docx]

**Table S10. Pearson correlations of perceived EE with symptoms (Sample 3; n=93).**

|  | **Patients’ perceived EE** | | |
| --- | --- | --- | --- |
|  | **Perceived criticism** | **Perceived EOI** | **Perceived warmth** |
| **Patients’ symptoms (PANSS)** |  |  |  |
| Positive symptoms | 0.21* | 0.17^+^ | -0.07 |
| Paranoia | 0.24* | 0.06 | -0.22 |

EE: Expressed Emotion; EOI: Emotional Over-Involvement; PANSS: Positive and Negative Syndrome Scale.

+p <.07; *p*<*0.05
