# Supplementary material for: The impact of family environment on self-esteem and symptoms in early psychosis
Source: PLoS One. 2021 Apr 5;16(4):e0249721. doi: 10.1371/journal.pone.0249721 (PMC8021173; doi:10.1371/journal.pone.0249721)
Supplement: S11 Table — (DOCX) [file pone.0249721.s012.docx]

**Table S11. Pearson correlations of patients’ SE with patients’ perceived EE and patients’ symptoms (Sample 3; n=93).**

|  | **Patients’ SE** | |
| --- | --- | --- |
|  | **Positive SE** | **Negative SE** |
| **Patients’ perceived EE (BDSEE)** |  |  |
| Perceived criticism | -0.22* | 0.25* |
| Perceived EOI | -0.21* | 0.29** |
| Perceived warmth | 0.21* | -0.18 |
| **Patients’ symptoms (PANSS)** |  |  |
| Positive symptoms | -0.05 | 0.21* |
| Paranoia | -0.18 | **0.34**** |

SE: Self-Esteem; EE: Expressed Emotion; BDSEE: Brief Dyadic Scale of Expressed Emotion; EOI: Emotional Over-Involvement; PANSS: Positive and Negative Syndrome Scale.

*p*<*0.05; **p*≤* 0.01. Medium effect sizes (r ≥ 0.30) in bold.
