# Supplementary material for: The impact of family environment on self-esteem and symptoms in early psychosis
Source: PLoS One. 2021 Apr 5;16(4):e0249721. doi: 10.1371/journal.pone.0249721 (PMC8021173; doi:10.1371/journal.pone.0249721)
Supplement: S12 Table — (DOCX) [file pone.0249721.s013.docx]

**Table S12. Conditional indirect effects of relatives’ EE on symptoms through perceived EE (M1) and negative SE (M2) (Sample 2; n=58).**

|  | **Conditional indirect effects at different values of the moderator** | | | | | | **Global Index of moderated mediation** | | | |
| --- | --- | --- | --- | --- | --- | --- | --- | --- | --- | --- |
|  |  |  |  |  | **95 % Bias-corrected**  **CI** | |  |  | **95 % Bias-corrected**  **CI** | |
| **SMM** | **Moderator** | **Level** | **Raw Parameter Estimate** | **SE** | **Lower** | **Upper** | **Index** | **SE** | **Lower** | **Upper** |
| **SMM1:**  Relatives’ Criticism🡪 Perceived Criticism🡪 Negative SE🡪 Positive Symptoms | Group | ARMS | 0.012 | 0.015 | -0.005 | 0.050 | 0.025 | 0.045 | -0.059 | 0.115 |
|  |  | FEP | 0.037 | 0.042 | -0.037 | 0.125 |  |  |  |  |
| **SMM2:**  Relatives Criticism🡪 Perceived Criticism🡪 Negative SE🡪 Paranoia | Group | ARMS | 0.007 | 0.006 | -0.002 | 0.022 | 0.006 | 0.017 | -0.030 | 0.039 |
|  |  | FEP | 0.013 | 0.016 | -0.021 | 0.044 |  |  |  |  |
| **SMM3:**  Relatives’ EOI🡪 Perceived EOI🡪  Negative SE🡪 Positive Symptoms | Group | ARMS | 0.013 | 0.010 | -0.000 | 0.039 | -0.011 | 0.017 | -0.053 | 0.014 |
|  |  | FEP | 0.002 | 0.014 | -0.033 | 0.023 |  |  |  |  |
| **SMM4:**  Relatives’ EOI🡪 Perceived EOI🡪  Negative SE🡪 Paranoia | Group | ARMS | 0.016 | 0.021 | -0.017 | 0.068 | -0.012 | 0.040 | -0.109 | 0.049 |
|  |  | FEP | 0.004 | 0.034 | -0.084 | 0.056 |  |  |  |  |

SMM: Serial Mediation Model.

^a^Relatives’ Criticism, Relatives’ EOI (X-Independent variable) and Diagnostic Category (W-moderator) were mean centered prior to analyses.

Note: Results are based on 10,000 bias-corrected bootstrap samples.

*95% Confidence Interval does not include zero.
